# One-Year HbA1c Predicts Long-Term Pancreas Graft Survival Following SPK Transplantation: A US Population Cohort Study

**DOI:** 10.3389/ti.2025.14940

**Published:** 2025-08-06

**Authors:** Georgios Kourounis, Samuel J. Tingle, Angeles Maillo-Nieto, Caroline Wroe, Emily R. Thompson, Ruth Owen, Leonie van Leeuwen, Matthew Holzner, Vikram Wadhera, Mohammed Zeeshan Akhtar, Sander Florman, James Shaw, Steve White, Colin Wilson

**Affiliations:** ^1^ Translational and Clinical Research Institute, Newcastle University, Newcastle upon Tyne, United Kingdom; ^2^ NIHR Blood and Transplant Research Unit, Newcastle University and Cambridge University, Newcastle upon Tyne, United Kingdom; ^3^ Institute of Transplantation, The Freeman Hospital, Newcastle upon Tyne, United Kingdom; ^4^ Recanati/Miller Transplantation Institute, Mount Sinai Hospital, New York, NY, United States

**Keywords:** pancreas transplantation, simultaneous pancreas-kidney transplantation, glycosylated hemoglobin (HbA1c), graft survival, long-term outcomes

## Abstract

Understanding which factors shape long-term pancreas graft outcomes after the critical first year post-transplantation is an ongoing challenge. This study assesses one-year HbA1c as a predictor of subsequent pancreas graft survival. A retrospective cohort study was conducted using the UNOS registry on all simultaneous pancreas-kidney (SPK) transplants between 2017 and 2023. Regression models with multiple imputations for missing data were used to evaluate predictors of long-term function. Non-linear relationships were modelled with restricted cubic splines (RCS). Among 2,917 SPK recipients (median follow-up 44 months, IQR: 25–60), one-year HbA1c was the strongest independent predictor of long-term graft survival. An HbA1c of 6.8% versus 4.4% (95th vs. 5th percentile) was associated with significantly worse graft survival (aHR = 2.48, 95% CI: 1.72–3.58). Simulated trial sample size calculations found that detecting a statistically and clinically significant reduction in one-year HbA1c from 7% to 6.5% would require 65 patients per group, whereas detecting a reduction in one-year graft loss from 12% to 9% would require 1,631 patients per group. HbA1c at 1 year is a robust, continuous marker of long-term graft function and may serve as a feasible, objective surrogate endpoint in future clinical trials, enabling smaller, more efficient study designs to evaluate interventions.

## Graphical Abstract

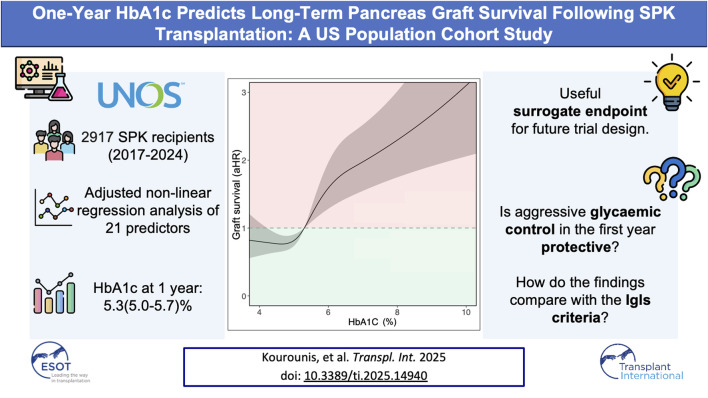

## Introduction

Diabetes has been described as a non-infectious pandemic disease of the modern era and is a leading cause of chronic kidney disease, non-traumatic lower limb amputations, and eye disease across the world [1–3]. Medical therapy in individuals with labile diabetes, even when optimized with hybrid closed loop insulin pump and continuous glucose monitoring systems, cannot restore optimal glycemic control with unavoidable continued exposure to low glucose levels and unremitting daily self-management burden, making β-replacement therapies the treatment of choice to restore long-term normoglycemia [4, 5]. In selected patients with diabetes-related kidney disease, simultaneous pancreas-kidney (SPK) transplantation offers optimal metabolic control, long-term insulin independence, fewer secondary complications, improved quality of life, and increased survival [2, 3, 6, 7].

While SPK transplantation has been shown to offer significant advantages, factors that can help predict long-term graft survival remain unclear. Previous registry analyses exploring predictors of graft survival have predominantly focused on donor and recipient factors at the time of transplantation. Factors linked with long-term graft survival have included donor age, donor cause of death, recipient and donor BMI, and cold ischemic times [3, 6]. These registry analyses have also shown that the first year post-transplant is critical, with graft loss occurring most frequently within the first year after transplant [2, 6, 8].

Among patients whose grafts continue to function beyond the first year, our understanding of the determinants of long-term outcomes remains limited. Moreover, little is known about modifiable factors that could guide post-transplant management and improve prognosis. With recipient HbA1c now routinely recorded in the UNOS dataset, there is an opportunity to investigate whether HbA1c at 1 year can serve as an independent predictor of subsequent graft survival in this cohort of patients. HbA1c is an intuitive candidate for this role. Given its routine use and potential to reflect ongoing pancreas function it has already been incorporated into post-transplantation scoring systems, such as the BETA-2 score [9, 10]. However, the independent association of HbA1c with long-term pancreas graft outcomes has not yet been clearly established. If proven to be predictive, HbA1c could serve as a clinically meaningful surrogate marker, informing patient management and guiding updates to clinical practice and transplant policy.

This study aimed to investigate whether one-year HbA1c serves as an independent predictor of long-term pancreas graft survival in SPK recipients whose grafts survived beyond the first year post-transplantation. The secondary objective was to evaluate the utility of one-year HbA1c as a surrogate marker of transplant outcomes, similar to the established role of one-year eGFR in kidney transplantation [11–13].

## Materials and Methods

### Study Design and Population

This population cohort study was conducted using data from the United Network for Organ Sharing (UNOS) Registry. Ethical review, approval, or informed consent specific to this study was not required as this was a secondary analysis of de-identified registry data. We included all recipients of SPK transplants between 1 January 2017, and 31 March 2023, with follow-up data available up to 31 March 2024. Exclusion criteria included recipients with graft loss within the first year post-transplant, patients with missing one-year HbA1c values, and those whose transplant indication was not diabetes. Although UNOS began recording follow-up HbA1c values in 2014, more than 98% of values were missing between 2014 and 2016, so data from those years were excluded. The study followed the Strengthening the Reporting of Observational Studies in Epidemiology (STROBE) reporting guidelines [14].

### Outcomes and Definitions

The primary outcome was recipient long-term graft survival. UNOS defines graft loss in pancreas transplantation as removal of the transplanted pancreas, recipient re-registered for pancreas or islet transplant, recipient returned to ≥0.5 units per kilogram per day of insulin for a duration of >90 days, or recipient death. At the time of data sharing UNOS confirmed that this definition had been in place since June 2015 and for the duration of this study.

UNOS records HbA1c values using the % Diabetes Control and Complications Trial (DCCT) units, where a value of 5.7% corresponds to 38.8 mmol/mol International Federation of Clinical Chemistry (IFCC) units. UNOS does not directly record center volume or follow-up eGFR. Instead, center volume was derived from the anonymized center identifiers in the registry by summing all SPK transplants performed at each center over the study period. The one-year follow-up eGFR values were calculated using the 2021 Chronic Kidney Disease-Epidemiology Collaborative (CKD-EPI) formula without race adjustment [15]. Median follow-up was estimated using the reverse Kaplan-Meier method with graft survival [16].

### Statistical Analysis

The approach to statistical analysis was similar to that described previously by our group [17]. Cox proportional hazards regression models were employed for the graft survival analyses. Multiple imputation was used to address missing data. The method used preserved non-linear relationships [18]. This was important as the outcome models employed non-linear modelling. For graft survival outcomes, we incorporated both the event indicator variable and the cumulative hazard of the event in the model to maintain the relationships between the outcome and the missing covariates [19].

Adjustment for a wide range of confounders was performed. Potential confounders were selected *a priori* based on prior research and clinical expertise [2, 3, 6, 20]. Statistical variable selection techniques (e.g., stepwise selection) were avoided [21]. A full list of covariates included along with justification for covariate selection, is provided in Supplementary Table S1. To account for potential non-linear relationships, restricted cubic splines were applied to continuous variables associated with the outcome. Three knots (10th, 50th, and 90th percentiles) were used for 1 year C-peptide, GFR, and recipient age, while four knots (5th, 35th, 65th, and 95th percentiles) were used for 1 year HbA1c to capture potential non-linearity while avoiding overfitting. An *a priori* decision was made to use splines for these variables. As transplanting center could impact post-transplant outcomes, we employed hierarchical modelling to adjust for this factor. This was done with a frailty Cox model, incorporating transplanting center as a random effect.

Acknowledging that donor and transplant factors might interact in ways that affect graft survival differently than when considered separately, we built models with interaction terms to account for these combined effects [22]. We also carried out sensitivity analyses to adjust for potential confounders excluded from the main models because of missing data or multicollinearity concerns [23].

Kaplan-Meier plots were generated to show crude graft survival, stratified by HbA1c levels <5.7% or ≥5.7%. This cutoff was selected based on previous literature [24] and the American Diabetes Association’s definition of a normal range (<5.7%) [25]. HbA1c was maintained as a continuous variable and was not stratified in the multivariable analyses.

To demonstrate the differing sample size requirements when using continuous outcomes versus binary outcomes, we simulated sample size calculations in R [26] with the ‘pwr’ [27] package. Assuming α = 0.05 and power = 0.80, our simulation varied effect sizes at 1 year post-transplant across a range of differences in graft loss incidence (binary outcome) and HbA1c values (continuous outcome). This range of effect sizes was selected to ensure that we captured all feasible minimally important differences. This approach enabled visualization and comparison of the sample sizes required.

We also conducted exploratory analyses on pancreas transplant alone (PA) and pancreas-after-kidney (PAK) recipients. Identical inclusion criteria, outcome definitions, and variable derivations were applied to these cohorts. Due to smaller sample sizes, only unadjusted Kaplan-Meier analyses were performed for PTA and PAK recipients.

Continuous variables were summarized using median and interquartile ranges. Outputs of models have been given as effect estimates with 95% confidence intervals. All analyses were performed in R (R Foundation for Statistical Computing, Vienna, Austria, version 4.4.1) [26], using the following packages; “tidyverse,” “finalfit,” “rms,” “Hmisc,” “survminer,” and “pwr” [27–32]. Plots were also generated in R using “ggplot2,” and “cowplot” [33, 34].

## Results

### Patient Demographics

**FIGURE 1:**
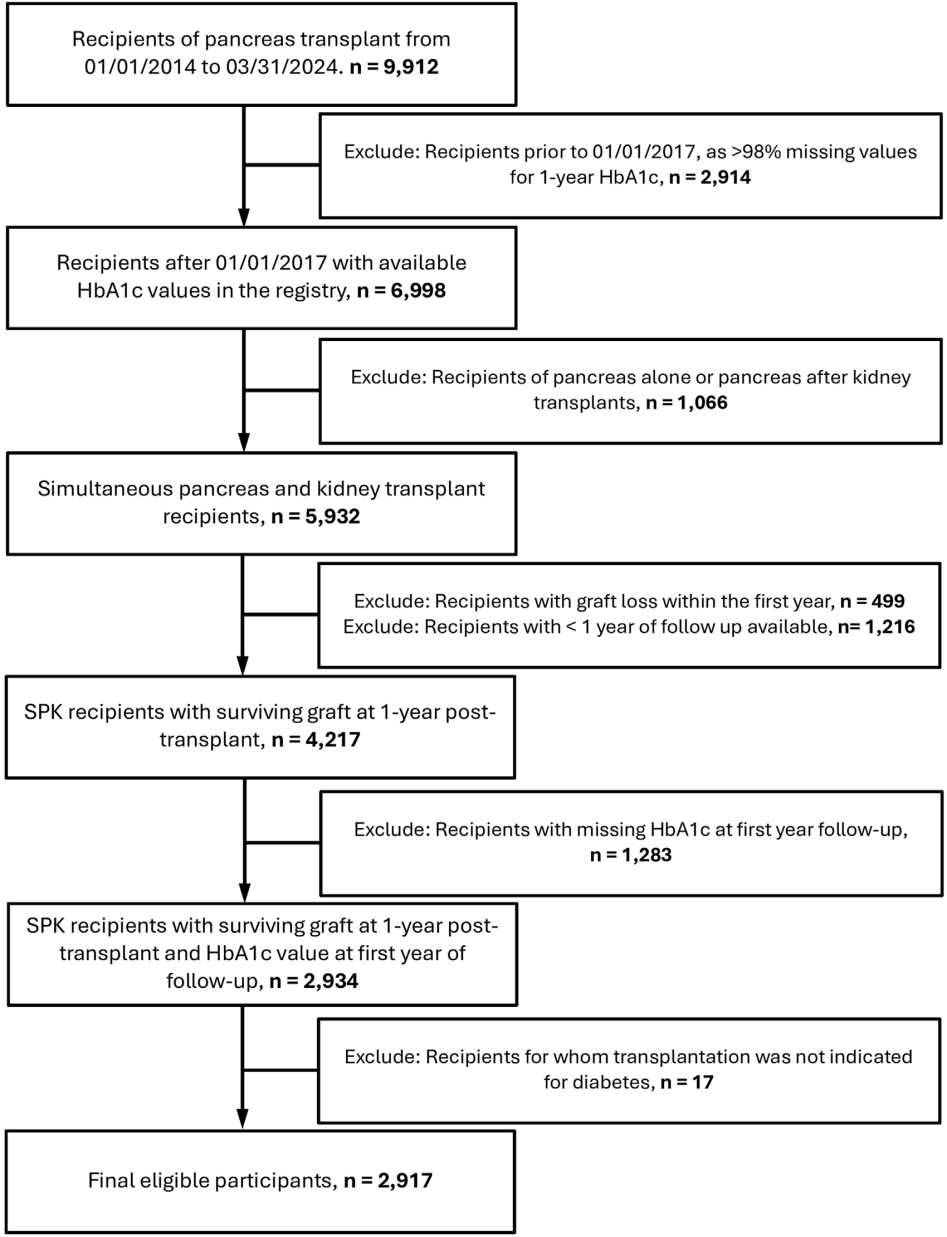
Flowchart of recipients included in the study.

From 2017 to 2023, a total of 5,153 SPK transplant recipients were identified, of which 2,917 met the inclusion criteria and had a functioning graft 1 year post-transplant. The process of exclusion and final cohort selection is outlined in the study flow diagram (Figure 1). Key cohort demographics are summarized in Table 1. Full demographic information, including missing data, is summarized in Supplementary Table S2.

**TABLE 1:** Donor and recipient demographic characteristics.

Variable	Levels	Values
Donor Age (years)	Median (IQR)	23.0 (18.0–30.0)
Donor Sex	Female	895 (30.7)
	Male	2022 (69.3)
Donor Ethnicity	White, Non-Hispanic	1714 (58.8)
	Black, Non-Hispanic	577 (19.7)
	Hispanic/Latino	512 (17.6)
	Asian, Non-Hispanic	67 (2.3)
	Other	47 (1.6)
Donor BMI (kg/m^2^)	Median (IQR)	23.6 (21.1–26.2)
Cause of Death	Head Trauma	1,535 (52.6)
	Drug overdose	437 (15.0)
	Other	945 (32.4)
Donor Type	DBD	2,827 (96.9)
	DCD	90 (3.1)
Recipient Age (years)	Median (IQR)	42.0 (35.0–49.0)
Recipient Sex	Female	1,123 (38.5)
	Male	1794 (61.5)
Recipient Ethnicity	White, Non-Hispanic	1,388 (47.6)
	Black, Non-Hispanic	830 (28.4)
	Hispanic/Latino	520 (17.8)
	Asian, Non-Hispanic	130 (4.5)
	Other	49 (1.7)
Recipient BMI (kg/m^2^)	Median (IQR)	25.7 (23.1–28.6)
Recipient Diabetes Type	Type 1	2,232 (76.5)
	Type 2	680 (23.3)
Waiting Time (days)	Median (IQR)	161.0 (50.0–421.0)
cPRA	≤20	2,361 (80.9)
	>20	553 (19.0)
Previous Pancreas Transplant	No	2,890 (99.1)
	Yes	27 (0.9)
Recipient Dialysis Status	No	659 (22.6)
	Yes	2,256 (77.4)
HLA Mismatch	≤2	102 (3.5)
	3	322 (11.0)
	4	755 (25.9)
	5	1,084 (37.2)
	6	654 (22.4)
CMV Match	P/N = Yes	748 (25.6)
	P/N = No	2,137 (73.3)
Duct Management	ED	2,723 (93.4)
	BD	86 (3.0)
	Other	108 (3.7)
Steroid Maintenance	0	805 (27.6)
	1	2007 (68.8)
Tacrolimus and MMF Maintenance	No	91 (3.1)
	Yes	2,795 (95.8)
HbA1c at 1 Year (%)	Median (IQR)	5.3 (5.0–5.7)
Treatment for Pancreas Rejection in 1st Year	No	2,230 (76.5)
	Yes	199 (6.8)
eGFR at 1 Year	Median (IQR)	71.2 (57.8–87.3)
Treatment for Kidney Rejection in 1st Year	No	2,251 (77.2)
	Yes	170 (5.8)

BD, Bladder drainage; BMI, Body mass index; cPRA, Calculated panel reactive antibody; CMV, Cytomegalovirus; DBD, Donation after brain death; DCD, Donation after circulatory death; ED, Enteric drainage; eGFR, Glomerular filtration rate; HbA1c, Glycosylated hemoglobin; HLA, Human leukocyte antigen; HTK, Histidine-Tryptophan-Ketoglutarate; IQR, Interquartile range; MMF, Mycophenolate mofetil; P/N, Donor positive, recipient negative; SKP, Simultaneous kidney-pancreas transplantation; UW, University of Wisconsin solution.

**FIGURE 2:**
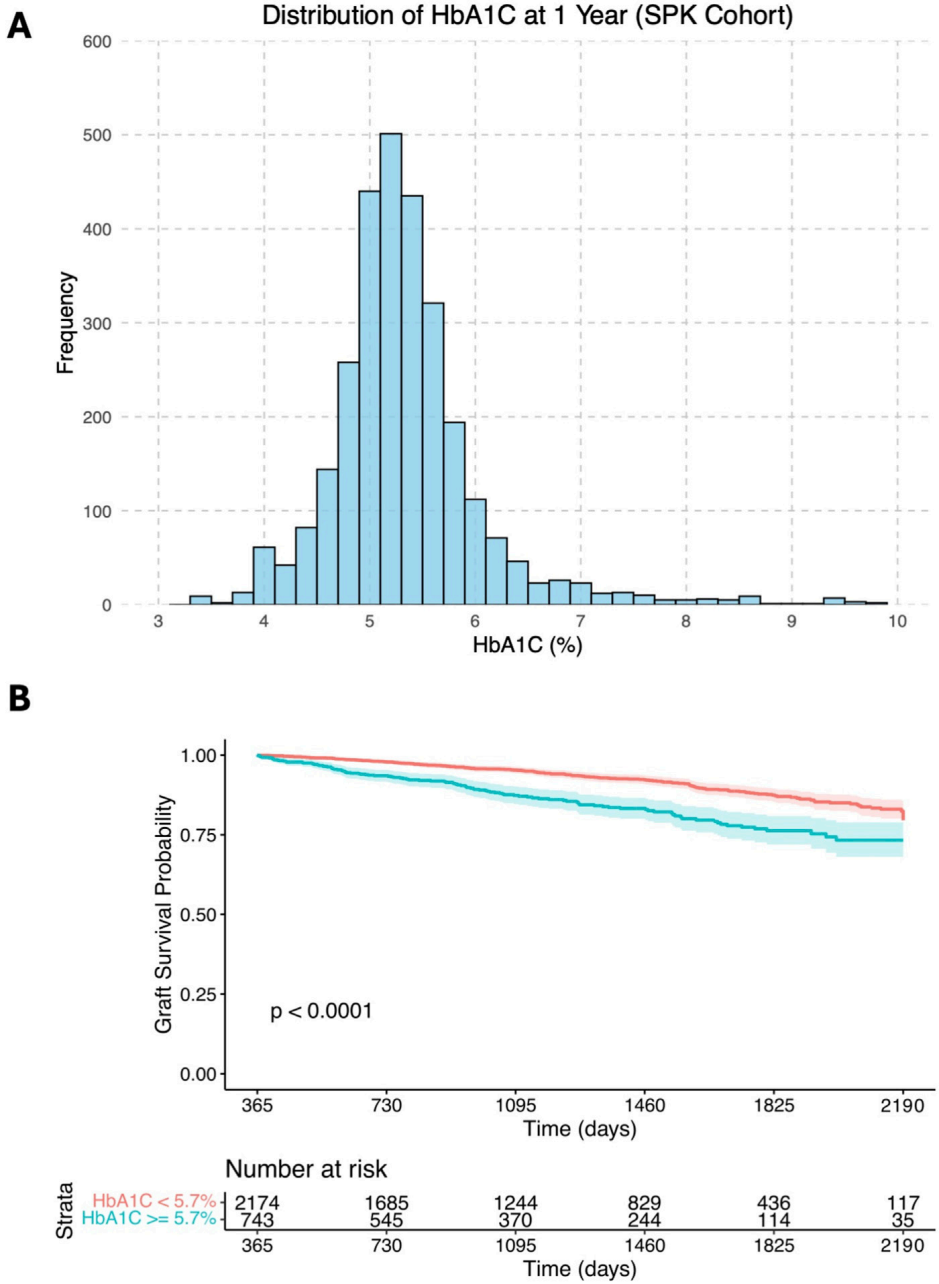
**(A)** Histogram showing the distribution of 1-year HbA1c levels in the study cohort; **(B)** Kaplan-Meier survival curves illustrating crude univariable long-term graft survival over days post- transplantation (starting from 1 year after transplantation), stratified by HbA1c levels of <5.7% and ≥5.7%.

The median 1-year HbA1c was 5.3% (IQR: 5.0%–5.7%), with its distribution shown in Figure 2A. The median follow-up time from transplantation, determined using the reverse Kaplan-Meier method, was 44 months (IQR: 25–60 months). Kaplan-Meier analysis (Figure 2B) illustrates crude univariable 5-year graft survival stratified by HbA1c levels (<5.7% vs. ≥5.7%).

### HbA1c as a Predictor of Long-Term Graft Survival in SPK

**FIGURE 3:**
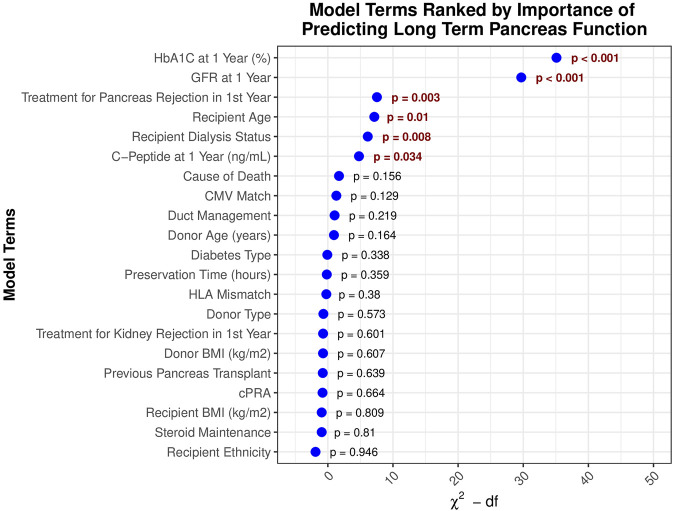
Model terms ranked by significance in predicting long-term pancreas graft survival.

**FIGURE 4:**
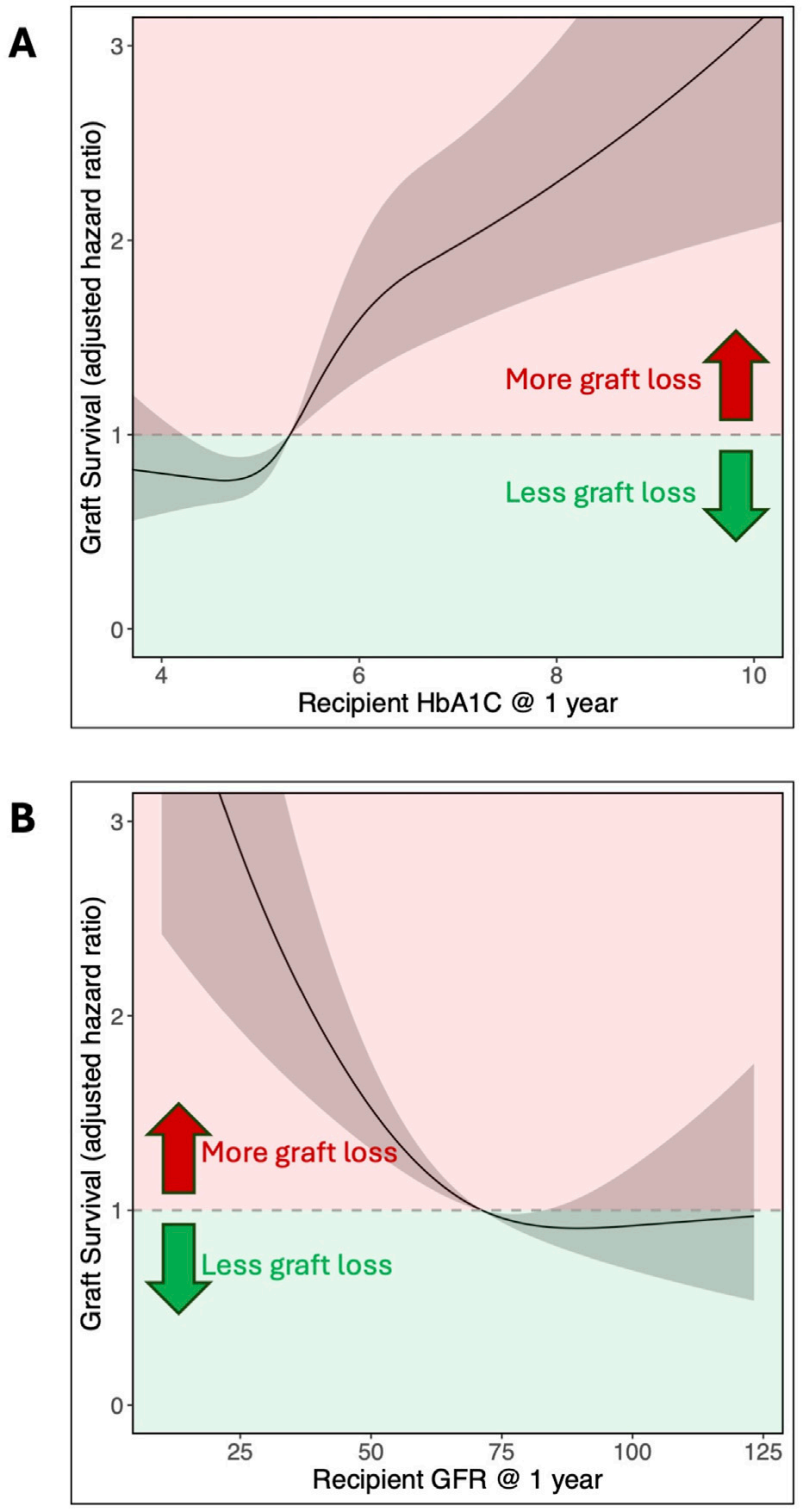
Adjusted hazard ratio for pancreas graft survival based on recipient **(A)** HbA1c (% DCCT) and **(B)** eGFR (mL/min/1.73 m^2^) at 1 year. The non-linear relationship is shown with restricted cubic splines analysis. HbA1c was modeled with 4 knots, while eGFR used 3 knots.

Multivariable Cox regression model analysis was used to assess the association of recipients’ HbA1c value at 1 year with long-term graft survival, adjusting for a wide range of factors (Table 2). Of these variables, one-year HbA1c was the strongest predictor of graft survival after 1 year (Figure 3), with its non-linear relationship confirmed in RCS analysis (p < 0.001, Figure 4A). One-year eGFR was the second strongest predictor of graft survival (Figure 3), with a non-linear relationship also confirmed in RCS analysis (<0.001, Figure 4B). Recipient age and one-year C-Peptide restricted cubic spline terms were visualized in Supplementary Figure S1.

**TABLE 2:** Multivariable Cox regression model for long-term graft survival, pooled from 20 imputed datasets. For restricted cubic splines see Figure 4 and Supplementary Figure S1.

Variable	Hazard ratio (95% CI)	P value
Donor age	1.011 (0.996–1.026)	0.164
Donor BMI	0.991 (0.960–1.024)	0.607
Donor cause of death		
Head trauma	Ref	
Drug overdose	0.860 (0.601–1.231)	0.411
Other	0.763 (0.577–1.008)	0.057
Donor type		
DBD	Ref	
DCD	1.218 (0.613–2.418)	0.573
Pancreas preservation time	0.986 (0.956–1.016)	0.359
HLA Mismatch	0.950 (0.848–1.065)	0.380
CMV Match		
P/N = Yes	Ref	
P/N = No	0.812 (0.620–1.062)	0.129
Duct management		
Enteric Drainage	Ref	
Bladder Drainage	1.608 (0.920–2.809)	0.095
Other	1.171 (0.688–1.995)	0.561
Steroids maintenance		
Yes	Ref	
No	0.966 (0.732–1.276)	0.810
Donor ethnicity		
Black	Ref	
White	0.996 (0.734–1.351)	0.979
Other	0.946 (0.664–1.350)	0.761
Recipient BMI	1.004 (0.972–1.038)	0.809
cPRA		
<20	Ref	
≥20	1.069 (0.790–1.447)	0.664
Recipient diabetes type		
Type 1	Ref	
Type 2	1.170 (0.849–1.612)	0.338
Previous pancreas transplant recipient		
No	Ref	
Yes	0.706 (0.165–3.016)	0.639
Recipient on dialysis		
No	Ref	
Yes	1.545 (1.123–2.126)	0.008
Treated for pancreas rejection in first year		
No	Ref	
Yes	1.744 (1.201–2.532)	0.003
Treated for kidney rejection in first year		
No	Ref	
Yes	1.118 (0.736–1.697)	0.601
RCS: Recipient age	RCS terms	0.010
RCS: HbA1c at first year follow-up	RCS terms	<0.001
RCS: C-Peptide at first year follow-up	RCS terms	0.034
RCS: eGFR at first year follow-up	RCS terms	<0.001

BMI, Body mass index; cPRA, Calculated panel reactive antibody; CMV, Cytomegalovirus; DBD, Donation after brain death; DCD, Donation after circulatory death; eGFR, Glomerular filtration rate; HbA1c, Glycosylated hemoglobin; HLA, Human leukocyte antigen; P/N, Donor positive, recipient negative; RCS, Restricted cubic splines.

In this cohort of patients who had survived with a functioning graft for at least 1 year, a one-year HbA1c of 6.8% (50.8 mmol/mol) at the 95th percentile compared to 4.4% (24.6 mmol/mol) at the 5th percentile was linked to poorer graft survival after 1 year (aHR = 2.48, 95% CI 1.72–3.58). Adjusting for Cox model covariates, an HbA1c of 5.0% (31.1 mmol/mol), which was the median in this cohort, resulted in a five‐year post-transplant graft loss probability of 6.4% (95% CI 2.8%–9.9%). This increased to 10.5% (4.7%–15.9%) for an HbA1c of 5.7% (38.8 mmol/mol), the third quartile for this cohort. Among the Igls criteria thresholds, an HbA1c of 6.5% (47.5 mmol/mol) yielded a predicted graft loss of 13.8% (6.0%–20.9%), and an HbA1c of 7.0% (53.0 mmol/mol) resulted in a predicted graft loss of 14.8% (6.5%–22.4%). A full summary of predicted graft loss by HbA1c level at 2–5 years post-transplant, adjusted for Cox model covariates, is provided in Table 3.

**TABLE 3:** Predicted graft loss by HbA1c levels at 2–5 years post‐transplant, adjusted for Cox model covariates.

HbA1c level (% DCCT/mmol/mol IFCC)	Predicted graft loss (% & 95% Confidence interval)			
	2 years	3 years	4 years	5 years
4.4%/24.6 mmol/mol	1.1% (0.4%–1.8%)	2.5% (1%–3.9%)	3.8% (1.5%–6%)	6.1% (2.5%–9.5%)
5.0%/31.1 mmol/mol	1.2% (0.5%–1.9%)	2.6% (1.1%–4.1%)	4% (1.7%–6.2%)	6.4% (2.8%–9.9%)
5.3%/34.4 mmol/mol	1.5% (0.6%–2.3%)	3.2% (1.4%–4.9%)	4.9% (2.2%–7.5%)	7.8% (3.5%–11.8%)
5.7%/38.8 mmol/mol	2% (0.8%–3.2%)	4.3% (1.9%–6.7%)	6.6% (2.9%–10.1%)	10.5% (4.7%–15.9%)
6.5%/47.5 mmol/mol	2.7% (1.1%–4.3%)	5.7% (2.4%–9%)	8.7% (3.7%–13.4%)	13.8% (6%–20.9%)
7.0%/53.0 mmol/mol	2.9% (1.1%–4.6%)	6.2% (2.6%–9.6%)	9.4% (4%–14.5%)	14.8% (6.5%–22.4%)
8.0%/63.9 mmol/mol	3.4% (1.3%–5.4%)	7.1% (2.9%–11.2%)	10.8% (4.5%–16.7%)	17% (7.3%–25.7%)

Cox model adjusted for: donor age, donor BMI, donor cause of death, donor type, pancreas preservation time, HLA mismatch, CMV match, duct management strategy, steroid maintenance, donor ethnicity, recipient age, recipient BMI, cPRA, recipient diabetes type, prior pancreas transplant, dialysis at transplant, treatment for pancreas rejection in the first year, treatment for kidney rejection in the first year, C-peptide at one-year follow-up, HbA1c at one-year follow-up, and eGFR at one-year follow-up.

To adjust for potential individual transplant center effects, a Cox frailty model with random effects for transplant center was performed, revealing no significant variation between transplant centers (P = 0.324, χ^2^ = 1.949, df = 1.756). When adjusting for this between-center variation, the RCS estimates for the impact of HbA1c on graft survival did not meaningfully change.

Sensitivity analyses were carried out using additional Cox proportional hazards models that incorporated factors such as donor age, ethnicity, smoking history, terminal lipase, time from admission to death, recipient waiting time, induction medications, center volume, and preservation fluid. An additional analysis with a death-censored graft failure endpoint was also carried out (Supplementary Table S3; Supplementary Figure S2). None of these analyses altered the conclusions derived from the main model findings.

Formal interaction assessments were conducted to check if the impact of HbA1c on graft survival was different along different levels of eGFR, or whether the recipient had received any insulin during the first year post-transplant. There were no associations between HbA1c and eGFR (p = 0.408), or HbA1c and insulin use (p = 0.636). Overall, this further supports that HbA1c is an independent predictor of long-term graft loss across all levels of these factors.

### Utility of HbA1c as a Surrogate Marker of Outcome

As HbA1c at 1 year was confirmed to be the strongest predictor of long-term graft survival, we sought to assess its utility as a surrogate marker for transplant outcomes in the context of clinical trial design. Our simulations revealed that a clinical trial using incidence of graft loss as its primary outcome, powered to detect a relative risk reduction of at least 25%—i.e. an absolute risk reduction from 12% to 9%—would require a total sample size of 3,262. In contrast, if the continuous HbA1c was used as a primary outcome measure, detecting a reduction of at least 0.5% (for example, from 7% to 6.5%) would require a total sample size of 130.

**FIGURE 5:**
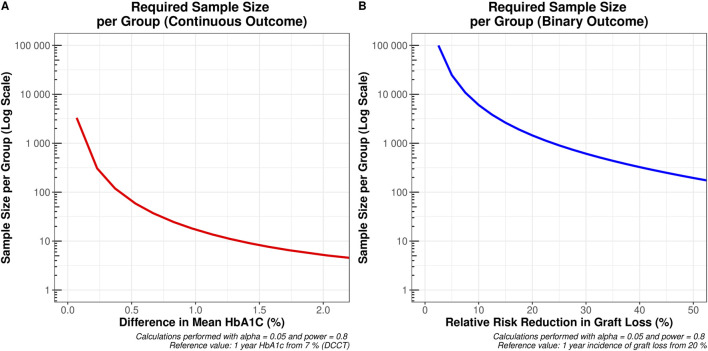
Sample sizes required to detect the desired minimum clinically important difference across the range of values on the x-axis **(A)** a change in one-year mean HbA1c from 7% (e.g., a reduction from 7% to 6.5% would require 65 patients per group) and **(B)** a relative risk reduction in graft loss from 20% at 1 year (e.g., a reduction from 20% to 16% would require 1,444 patients per group).

Figure 5 illustrates the required sample sizes across a range of possible minimum effect sizes for both the continuous HbA1c, and the binary relative risk of graft loss at 1 year. Due to the considerable difference in sample sizes between these outcomes, a log-transformed y-axis was necessary to effectively visualize these trends.

### Exploratory Analyses

Because of the limited number of PA and PAK recipient outcomes available in the UNOS registry since HbA1c data collection began, a comparable multivariable analysis was not possible. We have summarized the donor and recipient characteristics for these cohorts in Supplementary Table S4. Exploratory univariable analyses using Kaplan–Meier plots yielded similar results for the PA cohort, while no significant differences in long-term graft survival were observed in the PAK cohort (Supplementary Figures S3 and S4).

In the primary analysis, no significant association was observed between C-Peptide levels below 5 ng/mL and graft loss. However, the hazard of graft loss increased at C-Peptide levels above 5 ng/mL (Supplementary Figure S1). To further explore this relationship, we conducted an interaction analysis to assess whether the relationship between C-Peptide and graft loss differed by insulin use (Supplementary Figure S5). In this assessment, among recipients receiving exogenous insulin, the risk of graft loss was high at lower C-Peptide levels (<3 ng/mL). In contrast, for those not receiving exogenous insulin, the risk of graft loss was high at higher C-Peptide levels (>5 ng/mL). Associations outside these ranges were not significant.

We also applied our multivariable Cox-regression methodology to evaluate predictors of kidney-graft survival in the same cohort. As expected, eGFR emerged as the strongest predictor in this analysis, while HbA1c showed no association with graft outcome (Supplementary Figure S6).

## Discussion

Our findings demonstrate that one-year HbA1c was the strongest independent predictor of long-term graft survival in recipients of SPK transplants whose pancreas continued to function beyond the first year. The findings also highlight the potential utility of HbA1c as a primary endpoint in clinical trials, as it is a good surrogate for long-term graft survival. As a continuous measure, HbA1c could enable a substantial reduction in required sample sizes compared to binary measures of outcome.

This is also the first study to evaluate the association between one-year eGFR and subsequent pancreas graft survival in this population. One-year eGFR emerged as the second strongest independent predictor of pancreas graft outcomes, with lower eGFR values associated with an increased risk of pancreas graft loss. As this study includes only SPK recipients, reduced kidney function at 1 year likely reflects early rejection episodes affecting both organs, leading to subsequent pancreas graft loss.

These findings align with previous cohort studies that have explored the link between early metabolic assessments in pancreas transplant recipients and graft failure [9, 35–37]. Chetbourn et al. found that BETA-2 scores calculated 3 months post-transplantation in 209 pancreas transplant recipients were a marker of long term insulin independence [9]. Similarly, in an adjusted multivariable analysis of 210 pancreas transplant recipients, Mittal et al. found that oral glucose tolerance tests performed within 2 weeks of transplantation were the strongest independent predictor of graft failure [35]. Although we used different measures, our results similarly indicate that post-transplant glycemic control is the strongest predictor of long-term graft survival, even when adjusting for a range of other clinically relevant variables.

A significant strength of this study is the cohort size and methodology employed, including the use of a Cox frailty model to adjust for center-specific effects. This is particularly important given longstanding concerns among clinicians that pancreas graft loss may be reported inconsistently across centers. Although a standardized UNOS definition has been in place since June 2015, center-level variation in how outcomes are recorded or interpreted can still occur. These differences—whether due to follow-up protocols, thresholds for insulin restart, or documentation practices—can introduce variability in reported outcomes. By using a Cox frailty model, the analysis accounts for these unmeasured center-level differences. Importantly, the association between HbA1c and long-term graft survival remained even after this adjustment, strengthening the evidence that the finding is not affected by any potential inter-center variability.

In addition, multivariable analyses with interaction terms enabled us to control for potential confounders and assess whether associations varied across different variable levels. Kidney function was of particular interest, as renal impairment makes HbA1c interpretation more challenging and might synergistically influence graft survival with HbA1c. Our interaction analyses confirmed that the effect of HbA1c on graft outcomes remained consistent regardless of differences in eGFR levels–HbA1c was predictive of outcome even in those with low eGFR.

The results could significantly impact future pancreas transplantation research. Graft loss is becoming increasingly less prevalent and is a binary measure of outcome [2, 6, 8]. This combination poses significant challenges for powering clinical trials, as it necessitates large numbers of patients per group. In kidney transplantation, one-year eGFR has been adopted as an effective surrogate endpoint [11, 12]. HbA1c, a similarly continuous measure, also has the potential to serve as a surrogate marker in pancreas transplantation. Our findings demonstrate that HbA1c not only predicts long-term graft survival but also reduces the required sample size for adequately powered trials. Adopting HbA1c as a trial outcome could lower trial costs, speed up research timelines, and help bring effective interventions to clinical practice sooner.

In terms of identifying predictors of long-term pancreas survival, this is the first registry analysis to demonstrate how these predictors change over time. Previous registry analyses have only explored predictors of graft survival at the time of transplantation, reporting factors such as younger donor age, donor body mass index, and cause of death as important predictors of long-term graft survival [3, 6]. However, in recipients whose grafts survived up to the first year these factors became less important, with HbA1c and eGFR emerging as the strongest predictors. In other words, events during the first year post-transplant carry greater prognostic value than the donor and recipient characteristics present at transplantation.

This study is also the first to quantify the adjusted hazard ratio (aHR) of graft loss along a continuum of HbA1c values, made possible by the restricted cubic spline analysis. In our cohort of SPK recipients, the aHR for graft loss begins to increase at an HbA1c of 5.7%, which aligns with the American Diabetes Association’s cut-off for a normal range [25]. This contrasts to the Igls criteria for β-cell replacement therapy that defines optimal and good glycemic control at ≤6.5% and <7% HbA1c respectively [7, 38]. Our data suggest that these thresholds may be too high for SPK recipients. Three-quarters of recipients in our analysis had HbA1c values at or below 5.7% (the third quartile), with few recipients exceeding 6.5%. In addition, both the 6.5% and 7% thresholds fell within the range associated with significantly increased aHRs for graft loss (Figure 4).

In addition to evaluating the predictive value of one-year HbA1c for long-term graft survival, we also sought to explore whether its impact varied according to the recipients’ use of diabetes medications. Unfortunately, the registry data had significant gaps on recipients’ dosing and duration of oral hypoglycemic and insulin therapy post-transplant. This limited our ability to determine whether individuals with low HbA1c had better outcomes independently of medication use. Future studies should aim to capture more detailed information on medication use so as to evaluate whether targeted interventions to optimize glycemic control in the first year may lead to improved one-year and long-term pancreas outcomes. Supported by data showing the intrinsic impact of islet graft function on HbA1c regardless of exogenous insulin use, we propose that HbA1c is likely to be an important predictor even in those using glucose lowering medications [39].

The observed association between elevated C-peptide levels and graft loss was noteworthy. Although C-peptide concentrations below 5 ng/mL were not significantly associated with outcomes, levels exceeding this threshold were independently associated with an increased risk of graft failure. Interaction analysis further distinguished this to be the case only in those not receiving any exogenous insulin. This finding is not novel and aligns with prior reports linking elevated C-peptide with adverse graft outcomes [40, 41]. High C-Peptide in this cohort could reflect more insulin resistance or be an early marker of rejection. As the UNOS registry does not specify whether C-peptide measurements were taken in the fasting or stimulated state, these findings should be interpreted with caution. The absence of standardized collection highlights the need for more rigorous and consistent data capture for this key variable in future studies.

Another limitation of the UNOS registry is a lack of information on hemoglobin values at 1 year post transplantation. Hemoglobin is a potentially important confounder as anemia can make the interpretation of HbA1c more challenging. Analyzing the effect of anemia and adjusting for this was not possible with the current dataset.

Overall, these limitations also prevented us from comparing the Igls criteria for β-cell graft function to the UNOS graft failure definition. Key Igls components such as severe hypoglycemic episodes, insulin dosing, and reliably recorded fasting or stimulated C-peptide levels were not available in the UNOS registry. Future prospective studies should aim to capture these parameters to evaluate how different graft failure definitions impact observed event rates and trial design considerations.

Finally, we recognize that the results may not readily generalize to recipients of pancreas alone (PA) and pancreas after kidney (PAK) transplants. A robust multivariable analysis of this group was not possible due to the limited sample size. These exploratory findings are a call to action for further detailed studies across centers and improved data capture within the UNOS registry.

## Conclusion

One-year HbA1c was the strongest independent predictor of long-term graft survival in SPK recipients whose grafts survived beyond 1 year. This supports the potential utility of HbA1c as a surrogate endpoint for clinical trials, enabling more efficient study designs with smaller sample sizes. Moving forward, more granular and nuanced data are needed—whether through improved adherence to UNOS registry protocols or through collaborative efforts among individual centers—to determine whether targeted interventions during the first year can improve both one-year and long-term pancreas outcomes.

## Data Availability

The data analyzed in this study is subject to the following licenses/restrictions: The data that support the findings of this study are available from UNOS upon reasonable request. Requests to access these datasets should be directed to https://optn.transplant.hrsa.gov/data/view-data-reports/request-data/.

## Abbreviations

aHR, adjusted hazard ratio; CI, confidence interval; DBD, donation after brain death; DCCT, Diabetes Control and Complications Trial; DCD, donation after circulatory death; eGFR, estimated glomerular filtration rate; HbA1c, glycosylated hemoglobin; IFCC, International Federation of Clinical Chemistry; IQR, interquartile range; OPTN, Organ Procurement and Transplantation Network; PA, pancreas alone; PAK, pancreas after kidney; RCS, restricted cubic splines; SPK, simultaneous pancreas-kidney; UNOS, United Network for Organ Sharing.
