# Supplementary material for: One-Year HbA1c Predicts Long-Term Pancreas Graft Survival Following SPK Transplantation: A US Population Cohort Study
Source: Transpl Int. 2025 Aug 6;38:14940. doi: 10.3389/ti.2025.14940 (PMC12364725; doi:10.3389/ti.2025.14940)
Supplement: Supplementary file 1 [file DataSheet1.docx]

# **Supplementary file**

**One-Year HbA1c Predicts Long-Term Pancreas Graft Survival Following SPK Transplantation: A US Population Cohort Study**

Georgios Kourounis, Samuel J Tingle, Angeles Maillo-Nieto, Caroline Wroe, Emily R Thompson, Ruth Owen, Leonie van Leeuwen, Matthew Holzner, Vikram Wadhera, Mohammed Zeeshan Akhtar, Sander Florman, James Shaw, Steve White, Colin Wilson

**Capsule sentence summary & Graphical abstract**

HbA1c measured 12 months post-SPK was the most important predictor of subsequent pancreas-graft survival. It offers a practical continuous surrogate endpoint to be adopted in future pancreas transplantation trial design, similar to eGFR in kidney transplantation.


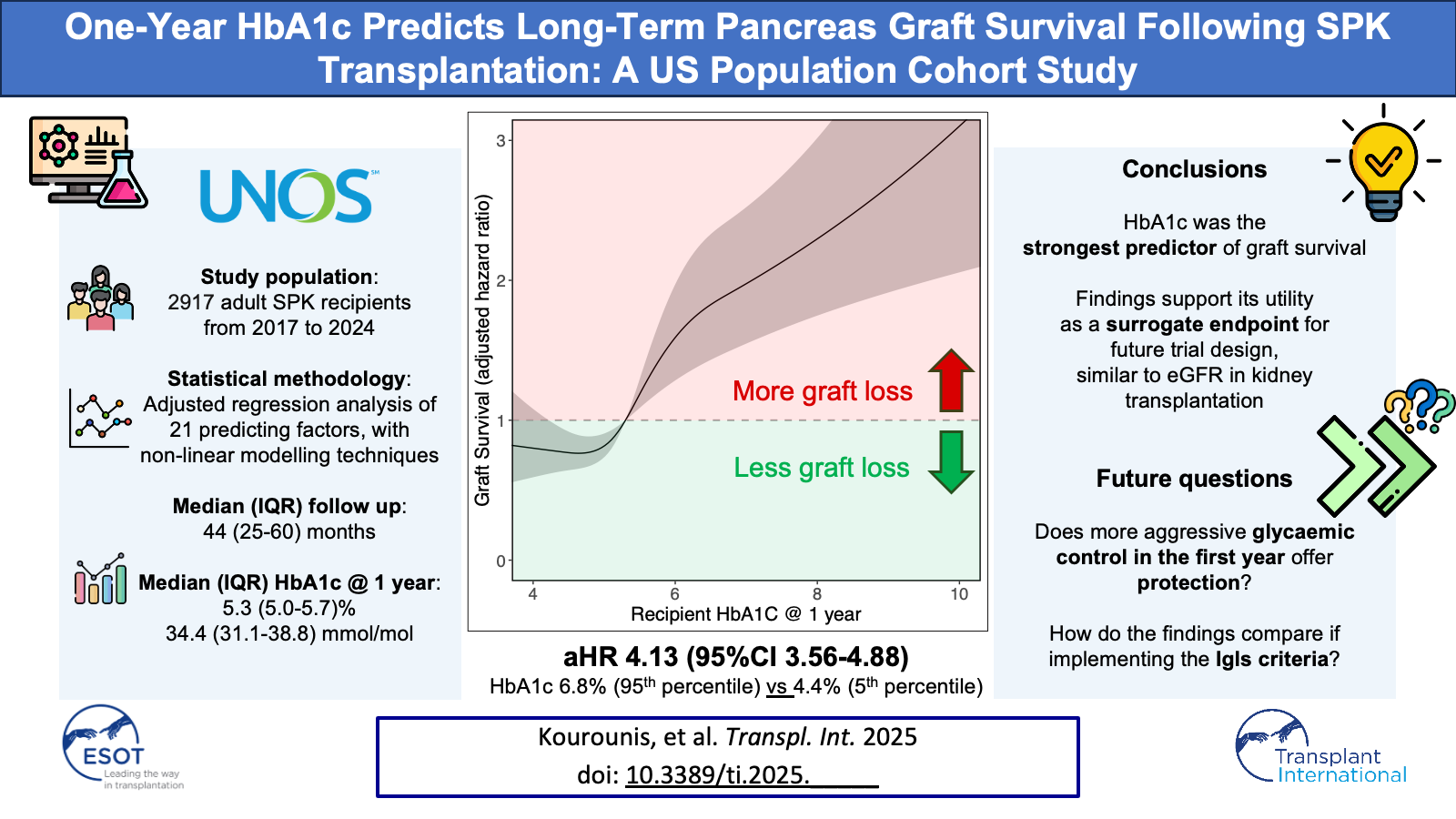


### **Supplementary File Contents**

**Supplementary Table 1**. Covariates included in the adjusted Cox regression model with justification and references.

**Supplementary Table 2**. Extended donor and recipient demographic characteristics.

**Supplementary Table 3**. Multivariable cox model with death-censored graft failure endpoint.

**Supplementary Table 4**. Extended donor and recipient demographic characteristics in the pancreas alone and pancreas after kidney cohorts.

**Supplementary Figure 1**. Associations between long-term graft survival and RCS terms from Table 2.

**Supplementary Figure 2**. Model terms ranked by significance in predicting long-term pancreas graft survival in the multivariable cox model with death-censored graft failure.

**Supplementary Figure 3**. Univariable Kaplan-Meier analysis of long-term graft survival, stratified by HbA1c for recipients of pancreas alone transplants.

**Supplementary Figure 4**. Univariable Kaplan-Meier analysis of long-term graft survival, stratified by HbA1c for recipients of pancreas after kidney transplants.

**Supplementary Figure 5**. Adjusted hazard ratio plots for graft survival against 1-year C-Peptide levels, comparing patients who received insulin in the first year versus those who did not.

**Supplementary Figure 6**. Model terms ranked by significance in predicting long-term kidney graft survival.

**Supplementary Figure 7**. Violin plots showing the distribution of HbA1c values at one year across various eGFR ranges.

**Supplementary Table 1.** Covariates included in the adjusted Cox regression model with justification and references.

| **Covariate** | **Justification or reference to previous registry analyses where it was shown to be significant** |
| --- | --- |
| Donor age | (1, 2, 3, 4) |
| Donor BMI | (1, 3) |
| Donor cause of death | (1, 2) |
| Donor type | Clinical: Known difference in outcomes between DCD and DBD donors |
| Donor ethnicity | (2) |
| Pancreas preservation time | (1, 2, 3) |
| HLA mismatch | (1) |
| CMV match | (2) |
| Duct management | (2) |
| Steroids maintenance | (1) |
| Recipient age | (1, 4) |
| Recipient BMI | (3, 4) |
| cPRA | (1, 2) |
| Recipient diabetes type | (1) |
| Previous pancreas transplant recipient | (3) |
| Recipient on dialysis | (1) |
| Treated for pancreas rejection in first year | (1, 2) |
| Treated for kidney rejection in first year | (1, 2) |
| C-Peptide at first year follow-up | Clinical: Marker of pancreas function |
| HbA1c at first year follow-up | Clinical: Marker of pancreas function |
| eGFR at first year follow-up | Clinical: Marker of kidney function that may affect the accuracy and interpretation of HbA1c values |
| **References:** (1) Gruessner AC, Gruessner RWG. The 2022 International Pancreas Transplant Registry Report—A Review. Transplant Proc. 2022 Sep 1;54(7):1918–43. (2) Gruessner AC, Gruessner RWG. Long-term outcome after pancreas transplantation: a registry analysis. Curr Opin Organ Transplant. 2016 Aug;21(4):377–85. (3) Miller G, Ankerst DP, Kattan MW, Hüser N, Stocker F, Vogelaar S, et al. Pancreas Transplantation Outcome Predictions—PTOP: A Risk Prediction Tool for Pancreas and Pancreas-Kidney Transplants Based on a European Cohort. Transplant Direct. 2024 May 15;10(6):e1632. (4) Gruessner AC, Gruessner RWG. The Current State of Pancreas Transplantation in the USA—ARegistry Report. Curr Transplant Rep. 2018 Dec 1;5(4):304–14. | |

**Supplementary** **Table 2**. Extended donor and recipient demographic characteristics.

| Variable | Levels | SKP | N Missing |
| --- | --- | --- | --- |
| **Donor Age (years)** | Median (IQR) | 23.0 (18.0 to 30.0) | 0 |
| **Donor Sex** | Female | 895 (30.7) | 0 |
|  | Male | 2022 (69.3) |  |
| **Donor Ethnicity** | White, Non-Hispanic | 1714 (58.8) | 0 |
|  | Black, Non-Hispanic | 577 (19.7) |  |
|  | Hispanic/Latino | 512 (17.6) |  |
|  | Asian, Non-Hispanic | 67 (2.3) |  |
|  | Other | 47 (1.6) |  |
| **Donor BMI (kg/m^2^)** | Median (IQR) | 23.6 (21.1 to 26.2) | 1 |
| **Cause of Death** | Head Trauma | 1535 (52.6) | 0 |
|  | Drug overdose | 437 (15.0) |  |
|  | Other | 945 (32.4) |  |
| **Donor History of Smoking** | N | 2784 (95.4) | 37 |
|  | Y | 96 (3.3) |  |
| **Donor Type** | DBD | 2827 (96.9) | 0 |
|  | DCD | 90 (3.1) |  |
| **Terminal Lipase (u/L)** | Median (IQR) | 29.0 (13.0 to 70.0) | 1 |
| **Donor Length of Stay (days)** | Median (IQR) | 5.0 (4.0 to 6.0) | 9 |
| **Recipient Age (years)** | Median (IQR) | 42.0 (35.0 to 49.0) | 0 |
| **Recipient Sex** | Female | 1123 (38.5) | 0 |
|  | Male | 1794 (61.5) |  |
| **Recipient Ethnicity** | White, Non-Hispanic | 1388 (47.6) | 0 |
|  | Black, Non-Hispanic | 830 (28.4) |  |
|  | Hispanic/Latino | 520 (17.8) |  |
|  | Asian, Non-Hispanic | 130 (4.5) |  |
|  | Other | 49 (1.7) |  |
| **Recipient BMI (kg/m^2^)** | Median (IQR) | 25.7 (23.1 to 28.6) | 0 |
| **Waiting Time (days)** | Median (IQR) | 161.0 (50.0 to 421.0) | 0 |
| **cPRA** | ≤20 | 2361 (80.9) | 3 |
|  | >20 | 553 (19.0) |  |
| **Diabetes Type** | Type 1 | 2232 (76.5) | 5 |
|  | Type 2 | 680 (23.3) |  |
| **Previous Pancreas Transplant** | N | 2890 (99.1) | 0 |
|  | Y | 27 (0.9) |  |
| **Recipient Dialysis Status** | N | 659 (22.6) | 2 |
|  | Y | 2256 (77.4) |  |
| **Preservation Time (hours)** | Median (IQR) | 9.5 (7.3 to 12.6) | 31 |
| **Pancreas Final Flush** | UW | 2120 (72.7) | 5 |
|  | HTK | 232 (8.0) |  |
|  | Other | 560 (19.2) |  |
| **HLA Mismatch** | ≤2 | 102 (3.5) | 0 |
|  | 3 | 322 (11.0) |  |
|  | 4 | 755 (25.9) |  |
|  | 5 | 1084 (37.2) |  |
|  | 6 | 654 (22.4) |  |
| **CMV Match** | P/N = Y | 748 (25.6) | 32 |
|  | P/N = N | 2137 (73.3) |  |
| **Duct Management** | ED | 2723 (93.4) | 0 |
|  | BD | 86 (3.0) |  |
|  | Other | 108 (3.7) |  |
| **Antibody Depletion Induction** | N | 1 (0.0) | 170 |
|  | Y | 2746 (94.1) |  |
| **Steroid Maintenance** | N | 805 (27.6) | 105 |
|  | Y | 2007 (68.8) |  |
| **Tacrolimus and MMF Maintenance** | N | 91 (3.1) | 31 |
|  | Y | 2792 (95.8) |  |
| **HbA1c at 1 Year (%)** | Median (IQR) | 5.3 (5.0 to 5.7) | 0 |
| **C-Peptide at 1 Year (ng/mL)** | Median (IQR) | 2.9 (2.1 to 4.3) | 1034 |
| **Insulin use in 1st Year** | N | 2475 (84.8) | 315 |
|  | Y | 127 (4.4) |  |
| **Treatment for Pancreas Rejection in 1st Year** | N | 2230 (76.5) | 488 |
|  | Y | 199 (6.8) |  |
| **eGFR at 1 Year** | Median (IQR) | 71.2 (57.8 to 87.3) | 10 |
| **Treatment for Kidney Rejection in 1st Year** | N | 2251 (77.2) | 496 |
|  | Y | 170 (5.8) |  |
| **BD** - Bladder drainage; **BMI** - Body mass index; **cPRA** - Calculated panel reactive antibody; **CMV** - Cytomegalovirus; **DBD** - Donation after brain death; **DCD** - Donation after circulatory death; **ED** - Enteric drainage; **eGFR** - Glomerular filtration rate; **HbA1c** - Glycosylated hemoglobin; **HLA** - Human leukocyte antigen; **HTK** - Histidine-Tryptophan-Ketoglutarate; **IQR** - Interquartile range; **MMF** - Mycophenolate mofetil; **N** - No; **P/N** – Donor positive, recipient negative; **SKP** - Simultaneous kidney-pancreas transplantation; **UW** - University of Wisconsin solution; **Y** - Yes. | | |  |

**Supplementary Table 3.** Multivariable cox model with death-censored graft failure endpoint.

| Variable | Hazard Ration  (95% Confidence Interval) | P value |
| --- | --- | --- |
| **AGE_DON** | 1.016 (0.993 to 1.040) | 0.173 |
| **BMI_DON_CALC** | 0.976 (0.928 to 1.027) | 0.349 |
| **COD_CAD_DON=Head Trauma** | Ref | - |
| **COD_CAD_DON=Drug overdose** | 0.670 (0.367 to 1.225) | 0.194 |
| **COD_CAD_DON=Other** | 0.760 (0.498 to 1.159) | 0.202 |
| **donor_type=DBD** | Ref | - |
| **donor_type=DCD** | 1.179 (0.360 to 3.867) | 0.785 |
| **PA_PRESERV_TM** | 0.971 (0.926 to 1.019) | 0.231 |
| **HLAMIS** | 0.891 (0.749 to 1.060) | 0.193 |
| **CMV_MATCH=P/N=Y** | Ref | - |
| **CMV_MATCH=P/N=N** | 0.714 (0.475 to 1.073) | 0.105 |
| **DUCT_MGMT=ED** | Ref | - |
| **DUCT_MGMT=BD** | 1.154 (0.358 to 3.717) | 0.810 |
| **DUCT_MGMT=Other** | 0.639 (0.226 to 1.808) | 0.398 |
| **STEROIDS_MAINT=0** | Ref | - |
| **STEROIDS_MAINT=1** | 1.784 (1.080 to 2.948) | 0.024 |
| **ETHCAT=Black** | Ref | - |
| **ETHCAT=Other** | 0.824 (0.491 to 1.381) | 0.462 |
| **ETHCAT=White** | 0.865 (0.546 to 1.371) | 0.538 |
| **BMI_CALC** | 1.026 (0.975 to 1.079) | 0.331 |
| **CPRA_CATEGORY=≤20** | Ref | - |
| **CPRA_CATEGORY=>20** | 1.275 (0.808 to 2.011) | 0.297 |
| **DIAB=Type I** | Ref | - |
| **DIAB=Type II** | 1.235 (0.744 to 2.052) | 0.414 |
| **PREV_PA_TX=N** | Ref | - |
| **PREV_PA_TX=Y** | 0.033 (0.000 to 2409.368) | 0.550 |
| **ON_DIALYSIS=N** | Ref | - |
| **ON_DIALYSIS=Y** | 1.891 (1.104 to 3.240) | 0.020 |
| **TRTREJ1Y_PA=N** | Ref | - |
| **TRTREJ1Y_PA=Y** | 2.149 (1.236 to 3.734) | 0.007 |
| **TRTREJ1Y_KI=N** | Ref | - |
| **TRTREJ1Y_KI=Y** | 1.245 (0.652 to 2.380) | 0.507 |
| **RCSoverallP: HbA1C_1year** | Wald test | <0.001 |
| **RCSoverallP: C_PEPTIDE_PA_1Y** | Wald test | 0.255 |
| **RCSoverallP: AGE** | Wald test | <0.001 |
| **RCSoverallP: GFR_1Y** | Wald test | 0.028 |

**Supplementary Table 4**. Extended donor and recipient demographic characteristics in the PA and PAK cohorts.

| Variable | Levels | PA | PAK |
| --- | --- | --- | --- |
| **Donor Age (years)** | Median (IQR) | 24.0 (18.0 to 30.0) | 22.0 (18.0 to 28.0) |
| **Donor Sex** | F | 71 (33.3) | 44 (22.3) |
|  | M | 142 (66.7) | 153 (77.7) |
|  | (Missing) | 0 (0.0) | 0 (0.0) |
| **Donor Ethnicity** | White, Non-Hispanic | 130 (61.0) | 126 (64.0) |
|  | Black, Non-Hispanic | 49 (23.0) | 41 (20.8) |
|  | Hispanic/Latino | 28 (13.1) | 28 (14.2) |
|  | Asian, Non-Hispanic | 2 (0.9) | 2 (1.0) |
|  | Other | 4 (1.9) | 0 (0.0) |
|  | (Missing) | 0 (0.0) | 0 (0.0) |
| **Donor BMI** | Median (IQR) | 23.1 (20.7 to 25.7) | 23.1 (20.3 to 25.8) |
| **Cause of Death** | Head Trauma | 94 (44.1) | 107 (54.3) |
|  | Drug overdose | 27 (12.7) | 20 (10.2) |
|  | Other | 92 (43.2) | 70 (35.5) |
|  | (Missing) | 0 (0.0) | 0 (0.0) |
| **Donor History of Smoking** | N | 202 (94.8) | 192 (97.5) |
|  | Y | 9 (4.2) | 4 (2.0) |
|  | (Missing) | 2 (0.9) | 1 (0.5) |
| **Donor Type** | DBD | 206 (96.7) | 196 (99.5) |
|  | DCD | 7 (3.3) | 1 (0.5) |
|  | (Missing) | 0 (0.0) | 0 (0.0) |
| **Terminal Lipase** | Median (IQR) | 35.0 (17.0 to 84.0) | 26.0 (14.0 to 59.0) |
| **Donor Length of Stay** | Median (IQR) | 5.0 (4.0 to 7.0) | 5.0 (4.0 to 6.0) |
| **Recipient Age** | Median (IQR) | 44.0 (35.0 to 52.0) | 43.0 (36.0 to 50.0) |
| **Recipient Sex** | F | 122 (57.3) | 92 (46.7) |
|  | M | 91 (42.7) | 105 (53.3) |
|  | (Missing) | 0 (0.0) | 0 (0.0) |
| **Recipient Ethnicity** | White, Non-Hispanic | 189 (88.7) | 135 (68.5) |
|  | Black, Non-Hispanic | 10 (4.7) | 30 (15.2) |
|  | Hispanic/Latino | 10 (4.7) | 29 (14.7) |
|  | Asian, Non-Hispanic | 2 (0.9) | 3 (1.5) |
|  | Other | 2 (0.9) | 0 (0.0) |
|  | (Missing) | 0 (0.0) | 0 (0.0) |
| **Recipient BMI** | Median (IQR) | 26.2 (23.5 to 29.7) | 25.8 (23.0 to 28.5) |
| **Waiting Time (days)** | Median (IQR) | 166.0 (58.0 to 387.0) | 436.0 (178.0 to 771.0) |
| **cPRA** | ≤20 | 153 (71.8) | 149 (75.6) |
|  | >20 | 60 (28.2) | 48 (24.4) |
|  | (Missing) | 0 (0.0) | 0 (0.0) |
| **Diabetes Type** | Type 1 | 206 (96.7) | 173 (87.8) |
|  | Type 2 | 5 (2.3) | 23 (11.7) |
|  | (Missing) | 2 (0.9) | 1 (0.5) |
| **Previous Pancreas Transplant** | N | 188 (88.3) | 159 (80.7) |
|  | Y | 25 (11.7) | 38 (19.3) |
|  | (Missing) | 0 (0.0) | 0 (0.0) |
| **Recipient Dialysis Status** | N | 0 (0.0) | 0 (0.0) |
|  | Y | 0 (0.0) | 0 (0.0) |
|  | (Missing) | 213 (100.0) | 197 (100.0) |
| **Transplant Date (years)** | Median (IQR) | 2.5 (1.3 to 4.0) | 2.5 (1.1 to 4.2) |
| **Center Volume** | Median (IQR) | 66.0 (51.0 to 214.0) | 36.0 (19.0 to 66.0) |
| **Preservation Time** | Median (IQR) | 10.2 (7.4 to 14.7) | 9.5 (6.8 to 13.2) |
| **PA Final Flush** | UW | 173 (81.2) | 139 (70.6) |
|  | HTK | 7 (3.3) | 21 (10.7) |
|  | Other | 33 (15.5) | 37 (18.8) |
|  | (Missing) | 0 (0.0) | 0 (0.0) |
| **HLA Mismatch** | ≤2 | 18 (8.5) | 11 (5.6) |
|  | 3 | 30 (14.1) | 24 (12.2) |
|  | 4 | 51 (23.9) | 45 (22.8) |
|  | 5 | 67 (31.5) | 67 (34.0) |
|  | 6 | 46 (21.6) | 50 (25.4) |
|  | (Missing) | 1 (0.5) | 0 (0.0) |
| **CMV Match** | P/N=Y | 57 (26.8) | 53 (26.9) |
|  | P/N=N | 153 (71.8) | 142 (72.1) |
|  | (Missing) | 3 (1.4) | 2 (1.0) |
| **Duct Management** | ED | 206 (96.7) | 192 (97.5) |
|  | BD | 3 (1.4) | 4 (2.0) |
|  | Other | 4 (1.9) | 1 (0.5) |
|  | (Missing) | 0 (0.0) | 0 (0.0) |
| **Antibody Depletion Induction** | No | 0 (0.0) | 0 (0.0) |
|  | Yes | 207 (97.2) | 179 (90.9) |
|  | (Missing) | 6 (2.8) | 18 (9.1) |
| **Steroid Maintenance** | 0 | 47 (22.1) | 45 (22.8) |
|  | 1 | 164 (77.0) | 141 (71.6) |
|  | (Missing) | 2 (0.9) | 11 (5.6) |
| **Tacrolimus and MMF Maintenance** | N | 5 (2.3) | 8 (4.1) |
|  | Y | 207 (97.2) | 186 (94.4) |
|  | (Missing) | 1 (0.5) | 3 (1.5) |
| **Pancreas graft survival time (years)** | Median (IQR) | 2.7 (1.0 to 4.0) | 2.1 (1.0 to 3.8) |
| **Patient survival time (years)** | Median (IQR) | 2.7 (1.0 to 4.0) | 2.2 (1.0 to 3.9) |
| **HbA1c at 1 Year** | Median (IQR) | 5.3 (5.0 to 5.6) | 5.3 (5.0 to 5.7) |
| **C-Peptide at 1 Year** | Median (IQR) | 2.4 (1.7 to 3.4) | 3.0 (2.1 to 4.1) |
| **Insulin Follow-up in 1st Year** | N | 194 (91.1) | 184 (93.4) |
|  | Y | 15 (7.0) | 8 (4.1) |
|  | (Missing) | 4 (1.9) | 5 (2.5) |
| **Treatment for PA Rejection in 1st Year** | N | 99 (46.5) | 155 (78.7) |
|  | Y | 31 (14.6) | 15 (7.6) |
|  | (Missing) | 83 (39.0) | 27 (13.7) |
| **GFR at 1 Year** | Median (IQR) | 69.3 (50.3 to 85.8) | 62.1 (48.3 to 75.8) |
| **Treatment for Kidney Rejection in 1st Year** | N | 0 (0.0) | 0 (0.0) |
|  | Y | 0 (0.0) | 0 (0.0) |
|  | (Missing) | 213 (100.0) | 197 (100.0) |
| **BD** - Bladder drainage; **BMI** - Body mass index; **cPRA** - Calculated panel reactive antibody; **CMV** - Cytomegalovirus; **DBD** - Donation after brain death; **DCD** - Donation after circulatory death; **ED** - Enteric drainage; **eGFR** - Glomerular filtration rate; **HbA1c** - Glycosylated hemoglobin; **HLA** - Human leukocyte antigen; **HTK** - Histidine-Tryptophan-Ketoglutarate; **IQR** - Interquartile range; **MMF** - Mycophenolate mofetil; **N** - No; PA – Pancreas alone; PAK – Pancreas after kidney; **P/N** – Donor positive, recipient negative; **SKP** - Simultaneous kidney-pancreas transplantation; **UW** - University of Wisconsin solution; **Y** - Yes. | | | |


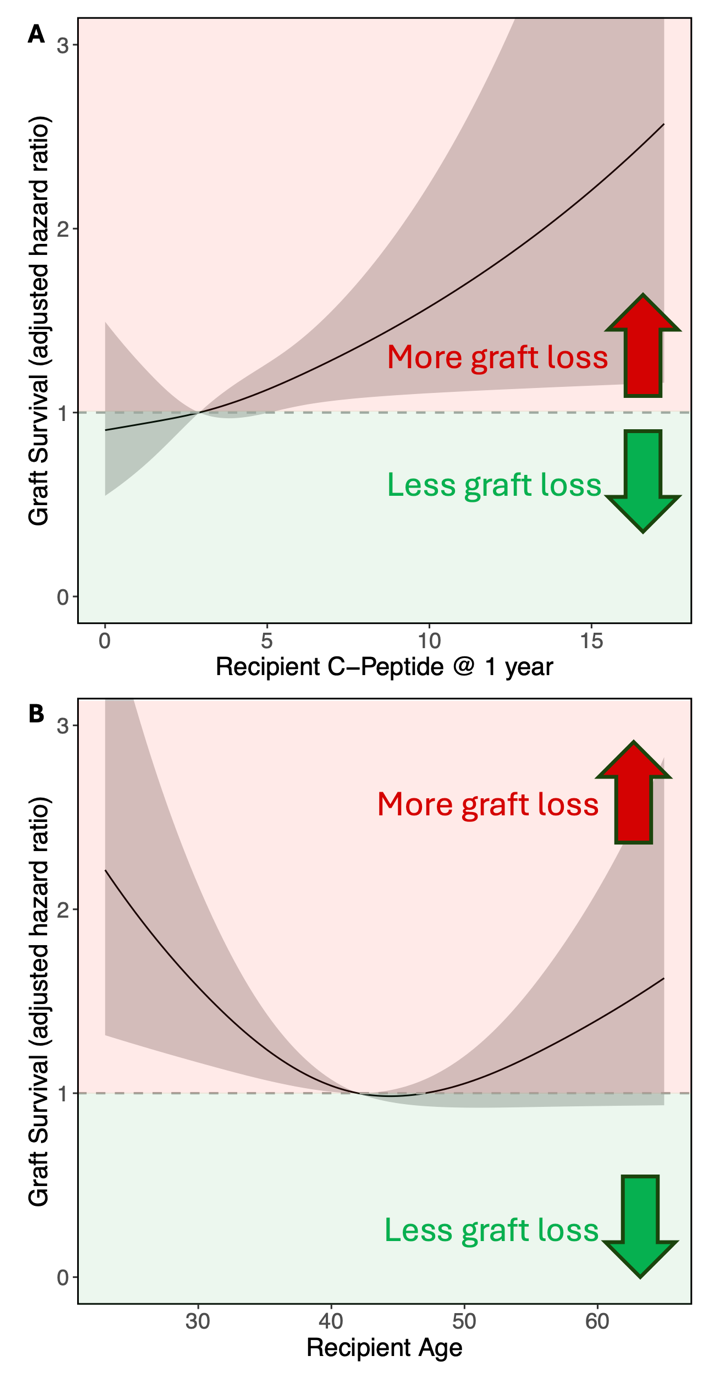


**Supplementary Figure 1**. Associations between long-term graft survival and: (A) recipient C-Peptide (ng/mL) at 1-year follow-up, (B) recipient age, visualized using restricted cubic splines. Associations modeled using 3 knots. These spline models are based on the model presented in Table 2.

~~~~

**Supplementary Figure 2**. Model terms ranked by significance in predicting long-term pancreas graft survival in the multivariable cox model with death-censored graft failure.

**Supplementary Figure 3**. Univariable Kaplan-Meier analysis of long-term graft survival over days post- transplantation (starting from 1 year after transplantation), stratified by HbA1c levels of <5.7% and ≥5.7% for recipients of pancreas alone transplants.

**Supplementary Figure 4**. Univariable Kaplan-Meier analysis of long-term graft survival over days post- transplantation (starting from 1 year after transplantation), stratified by HbA1c levels of <5.7% and ≥5.7% for recipients of pancreas after kidney transplants.

**Supplementary Figure 5**. Exploratory interaction analysis to assess whether the relationship between C-Peptide and graft loss differed by insulin use. Adjusted hazard ratio plots for graft survival by 1-year C-peptide levels, stratified by insulin use in the first year (orange = insulin users, green = non-users). The plots demonstrate a differential association between C-peptide levels and graft survival by insulin use. At lower C-peptide levels, non-insulin users exhibited lower rates of graft loss compared to insulin users with similar levels, with the difference diminishing at higher C-peptide levels. This figure and caption are intended to clarify the nature of the statistical interaction, without drawing clinical inferences.

**Supplementary Figure 6**. Model terms ranked by significance in predicting long-term kidney graft survival

**Supplementary Figure 7.** Violin plots showing the distribution of HbA1c values at one year across various eGFR ranges. Each violin illustrates the density and spread of HbA1c measurements within a given eGFR category.
